# Linking animal migration and ecosystem processes: Data‐driven simulation of propagule dispersal by migratory herbivores

**DOI:** 10.1002/ece3.9383

**Published:** 2022-10-18

**Authors:** Marius Somveille, Diego Ellis‐Soto

**Affiliations:** ^1^ Department of Biology Colorado State University Fort Collins Colorado USA; ^2^ Department of Genetics, Evolution and Environment, Centre for Biodiversity and Environment Research University College London London UK; ^3^ Department of Ecology and Evolutionary Biology Yale University New Haven Connecticut USA; ^4^ Center for Biodiversity and Global Change Yale University New Haven Connecticut USA

**Keywords:** animal migration, ecosystem process, Galapagos tortoises, migratory connectivity, seed dispersal

## Abstract

1. Animal migration is a key process underlying active subsidies and species dispersal over long distances, which affects the connectivity and functioning of ecosystems. Despite much research describing patterns of where animals migrate, we still lack a framework for quantifying and predicting how animal migration affects ecosystem processes. In this study, we aim to integrate animal movement behavior and ecosystem functioning by developing a predictive modeling framework that can inform ecosystem management and conservation.
2. We propose a framework to model individual‐level migration trajectories between populations' seasonal ranges as well as the resulting dispersal and fate of propagules carried by the migratory animals, which can be calibrated using empirical data at every step of the modeling process. As a case study, we applied our framework to model the spread of guava seeds, *Psidium guajava*, by a population of migratory Galapagos tortoises, *Chelonoidis porteri*, across Santa Cruz Island. Galapagos tortoises are large herbivores that transport seeds and nutrients across the island, while Guava is one of the most problematic invasive species in the Galapagos archipelago.
3. Our model can predict the pattern of spread of guava seeds alongside tortoises' downslope migration range, and it identified areas most likely to see establishment success. Our results show that Galapagos tortoises' seed dispersal may particularly contribute to guava range expansion on Santa Cruz Island, due to both long gut retention time and tortoise's long‐distance migration across vegetation zones. In particular, we predict that tortoises are dispersing a significant amount of guava seeds into the Galapagos National Park, which has important consequences for the native flora.
4. The flexibility and modularity of our framework allow for the integration of multiple data sources. It also allows for a wide range of applications to investigate how migratory animals affect ecosystem processes, including propagule dispersal but also other processes such as nutrient transport across ecosystems. Our framework is also a valuable tool for predicting how animal‐mediated propagule dispersal can be affected by environmental change. These different applications can have important conservation implications for the management of ecosystems that include migratory animals.

## Graphical abstract

We propose a framework to model animal migration trajectories and the resulting dispersal and fate of propagules carried by the migratory animals. Our model can predict the pattern of spread of guava seeds alongside the downslope migration range of Galapagos tortoises on Santa Cruz Island, and it identifies areas most likely to see establishment success. The flexibility and modularity of our framework allows for a wide range of applications to investigate how migratory animals affect ecosystem processes.

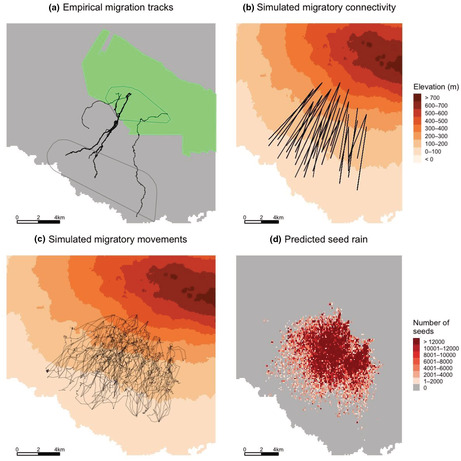

## 1 INTRODUCTION

Ecosystems are connected through flows of energy and matter transported passively by the abiotic environment or actively through the transport of individuals, gametes, or spores (Loreau et al., 2003). The movement of animals is a key process shaping active subsidies and dispersal of plants, which affects the functioning and connectivity of ecosystems (Côrtes & Uriarte, 2013; Earl & Zollner, 2017; Ellis‐Soto et al., 2021; Schmitz et al., 2018; Subalusky & Post, 2018). In particular, animal migration—the regular, directional movement of animals between specific destinations—involves billions of animals across the planet (Dokter et al., 2018; Hu et al., 2016) and provides important ecosystem services (Bauer & Hoye, 2014). However, despite the ecological importance of the migration phenomenon and the research efforts to describe patterns of where animals migrate, we still lack a predictive framework that we can use to quantify how migratory animals impact ecosystem functioning.

One of the main ecosystem services provided by migratory animals is propagule dispersal, particularly seeds (Bauer & Hoye, 2014). More than half of all plant species are dispersed by animals (Aslan et al., 2013), and such animal‐mediated seed dispersal influences plant species survival and range expansion into new environments (Kendrick et al., 2012; Nathan & Muller‐Landau, 2000; Travis et al., 2013). The movement ecology of seeds has thus been highlighted as a key knowledge gap to improve our understanding of plant distribution (Beckman et al., 2019). Quantifying and predicting the role of animals in seed dispersal is particularly relevant given that the scale of animal movements is declining globally in response to human activities (Tucker et al., 2021), which has important implications for plant–animal interactions (Neuschulz et al., 2016). Animal migrations, in particular, are disappearing at alarming rates (Wilcove & Wikelski, 2008) with unknown, but likely significant, consequences on ecosystem functioning. For instance, the loss of Pleistocene megafauna and their long‐ranging movements is thought to have significantly reduced seed dispersal across large spatial scales (Malhi et al., 2016; Pires et al., 2018).

Dispersal kernels, which allow modeling how far seeds can be dispersed, have been usually employed to understand the dispersal of plant species (Bullock et al., 2017; Nathan, 2006; Nathan et al., 2012; Pires et al., 2018). For animal‐mediated seed dispersal, however, it is important to take into account intraspecific variation in the seed dispersal ability of animals (Zwolak, 2018). In addition, modeling seed dispersal must ideally be spatially‐explicit in order to account for the context dependency of where seeds are deposited and the probability of successful germination and establishment (Nathan & Muller‐Landau, 2000). Developments in tracking technologies allow quantification of the movement of animals at fine spatiotemporal scales for long periods of time (Kays et al., 2015). This opens up opportunities to study animal movement and understand its underlying environmental and internal drivers (Hawkes et al., 2011; Jesmer et al., 2018; Nathan et al., 2008). Recent years have seen an increase in studies coupling GPS tracking of animals with ecosystem processes, especially seed dispersal, to provide a spatially‐explicit, individual‐based understanding of seed dispersal by animals that integrates movement, seeds dispersed, and gut retention times. Most of these studies modeled seed dispersal from daily foraging movements of resident animal populations (Campos‐Arveiz et al., 2008; Beaune et al., 2015; Kleyheeg et al., 2017; Olesky et al., 2017; Wright et al., 2020; van Toor et al., 2019), while Kleyheeg et al. (2019) modeled seed dispersal by a seasonally migrating local population of mallards. However, the latter only modeled dispersal from a single location, which limits the potential applications of this approach, and did not use empirical data explicitly to model the fate of the dispersed seeds once released in the environment.

Here we fill a gap in the literature by proposing a modeling framework that is data‐driven, spatially‐explicit, and multi‐population in order to simulate the spread of seeds by migratory herbivores. Our framework contrasts with previous work that modeled seed dispersal using spatially‐homogeneous dispersal kernels, daily foraging movement, or seasonal migration from a single location, and it allows us to model individual‐level migration trajectories between the seasonal distributions of multiple populations together with the resulting dispersal and fate of seeds carried by the migratory animals. In addition, our framework uses empirical data from a variety of sources to calibrate every step of the modeling process, thus going further than previous work that was partially data‐driven. Our approach aims to quantitatively harmonize behavioral ecology and ecosystem process, and make quantitative predictions that could inform ecosystem management and conservation. As a case study, we applied our framework to model the spread of guava seeds, *Psidium guajava* (Linnaeus), by a population of migratory Galapagos tortoises, *Chelonoidis porteri* (Rothschild) (IUCN critically endangered), across Santa Cruz Island. Galapagos tortoises are the largest terrestrial ectotherms worldwide and are considered ecosystem engineers through the transport of seeds and nutrients, as well as herbivory and trampling (Blake et al., 2012; Ellis‐Soto, 2020; Gibbs et al., 2010), while Guava is one of the most problematic invasive species in the Galapagos archipelago, having been introduced in the late 19th century (Walsh et al., 2008).

Santa Cruz Island contains the highest human population in the Galapagos. The island harbors more introduced plant species than native species (Guézou et al., 2010), which threatens endemic biodiversity. On Santa Cruz Island, the presence of invasive guava has modified the native plant community and ecosystems, especially so in the humid highlands (Guézou et al., 2010), and several ongoing eradication initiatives have so far proven unsuccessful (Gardener et al., 2010). Guava is heavily consumed by Galapagos tortoises (Blake et al., 2015), which perform seasonal migrations across the elevational gradient of the island, and disperse guava seeds throughout their range (Blake et al., 2012, 2013; Ellis‐Soto et al., 2017). Most of the island's surface is located within the Galapagos National Park (GNP) and surrounds heavily degraded agricultural land where invasive species such as guava are widespread (Benitez‐Capistros et al., 2019; Trueman et al., 2014). The permeability of the national park boundary to agricultural lands means that animals like tortoises regularly cross from the park into farmland during their seasonal migrations, where they consume introduced plants including fruits, before returning to the park (Benitez‐Capistros et al., 2018). Taking advantage of a long‐term study on Galapagos tortoises (Benitez‐Capistros et al., 2019; Blake et al., 2012, 2013, 2015; Sadeghayobi et al., 2011; Yackulic et al., 2016), we use data on tortoise ecology, migratory behavior, seed dispersal ability, diet preference and gut retention times in order to calibrate our modeling framework and simulate how tortoises spread guava across Santa Cruz island. Specifically, we aim to (i) simulate realistic migration trajectories for tortoises across the population's geographical range, (ii) model how guava seeds are spread by migrating tortoises, and (iii) use empirical data to calibrate the model and validate the seed dispersal predictions.

## 2 MATERIALS AND METHODS

### 2.1 Empirical data

#### 2.1.1 Study site and habitat

We obtained a landcover map for Santa Cruz Island, Galapagos, from (Rivas‐Torres et al., 2018) and shapefiles of the Galapagos National Park and agricultural land from the 2014 census conducted by the Ecuadorian Ministry of Agriculture (CGREG, 2015). This allowed us to estimate the proportion of different habitats where invasive guava is deposited through seed dispersal by *C. porteri* as well as the ratio of seed deposition in the national park and agricultural land to better understand the context dependency of these events. To estimate available resources for tortoises, we used the Normalized Difference Vegetation Index (NDVI), a remote sensing measure of greenness that correlates well with primary productivity, and which has been shown to be an important driver of tortoises’ annual migration (Bastille‐Rousseau et al., 2019; Blake et al., 2013; Yackulic et al., 2016). To estimate the spatial distribution of guava, we made use of a recently published land cover classification of the Galapagos, which provides the distribution of guava patches at very high resolution in Santa Cruz Island (Laso et al., 2019).

#### 2.1.2 Tortoise migratory movements

We used tracking data of 9 tagged adult male Galapagos giant tortoises for which hourly locations were recorded between 2010 and 2018 (attachment procedures and GPS sampling regimes are described in Blake et al., 2013). Galapagos tortoises of the species *C. porteri* (our study species) represent a partially migratory system, and in our dataset, a total of five adult male individuals performed altitudinal migrations during the time period of interest, while no clear downslope migratory movements could be identified for the other four in the time period of interest. To identify the starting and end locations of individual downslope migrations between 2010 and 2018, we made use of the locator() function from the graphics package in R on plots representing individual tortoise net square displacement (Singh et al., 2016), which provided us with 16,464 GPS tortoise locations. We obtained 17 downslope migration trajectories from the five individuals (i.e., individuals were tracked for several years; Figure S1).

#### 2.1.3 Tortoise diet and gut retention

Sampling of tortoise dung revealed that guava is the most common dispersed plant species by *C. porteri* with an average of 624 seeds per dung pile (Ellis‐Soto et al., 2017). Feeding trial experiments with pseudo seeds identified the gut retention time of *C. porteri* during the period of downslope migration for various plant species including guava (Sadeghayobi et al., 2011). To model gut retention time, we used a right‐skewed normal distribution with a skewness parameter of 2, a standard deviation of 4, and a mean of 12 days. The latter was taken from the results of feeding trial experiments with pseudo seeds that identified the gut retention time of *C. porteri* during the period of downslope migration for various plant species including guava (Sadeghayobi et al., 2011). In addition, ex situ germination trials suggest that tortoise ingestion and dung do not affect the germination success of guava seeds neither positively nor negatively (Blake et al., 2012).

### 2.2 Modeling framework

#### 2.2.1 Model overview

**FIGURE 1:**
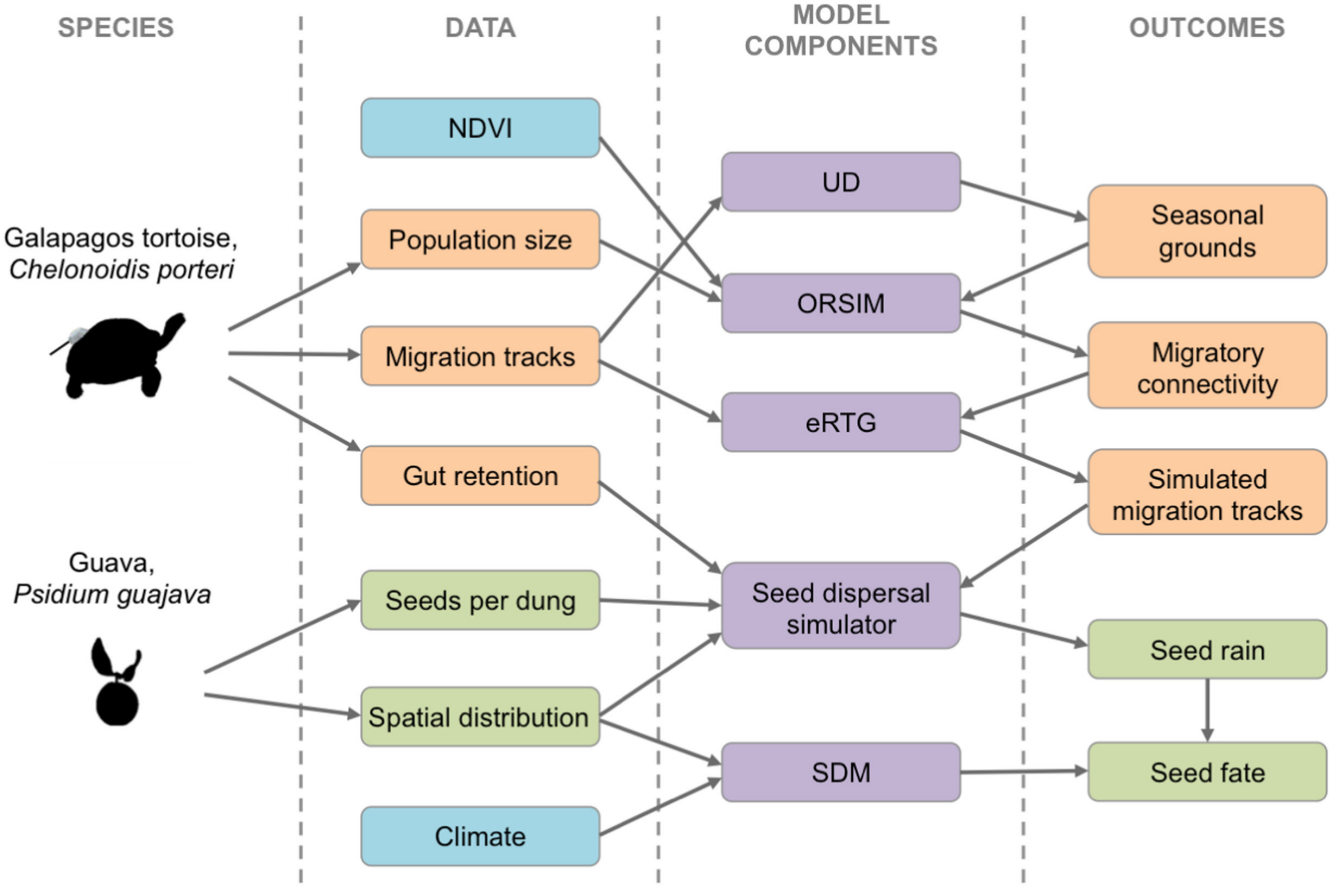
Workflow for data‐driven simulation of seed dispersal by a migrating species. Orange boxes indicate data and simulation outcomes for migratory Galapagos tortoises; green boxes indicate data and simulation outcomes for the invasive plant species guava; blue boxes indicate environmental data; and purple boxes indicate model components (see Material and Methods for details). NDVI—normalized difference vegetation index, a remote sensing measure capturing primary productivity; UD—utilization distributions, computed from empirical movement tracks; ORSIM—optimal redistribution simulator, which simulates migratory connectivity; eRTG—empirical random trajectory generator, which generates a movement between two endpoints that are calibrated by empirical migration tracks; SDM—species distribution model, indicating guava establishment success. Seed rain refers to the spatial distribution of the density of seeds resulting from seed dispersal by migrating tortoises; seed fate refers to the spatial distribution of the density of germinated seeds resulting from seed dispersal by migrating tortoises.

We simulated the migratory movement of tortoises from the highlands to the lowlands of Santa Cruz Island using a two‐step modeling process: First, we simulated the migratory connectivity of the population (i.e., links between seasonally‐occupied sites based on individual tortoises’ migratory movements), and second, we simulated the trajectory of migrating individuals (Figure 1).

#### 2.2.2 Simulating migratory connectivity

We simulated migratory connectivity between the seasonal distributions of tortoises, i.e., in the highlands and in the lowlands. To map areas seasonally utilized by tortoises, we calculated 95 percentile utilization distribution (UD; Fieberg & Kochanny, 2005) for each of the nine tortoises (see Section 2.1.2) using a kernel density estimator in the adehabitatHR package (Calenge, 2006). We then drew a geometric convex hull around these UDs to obtain a population‐level highland and lowland range. Highland and lowland areas were converted into presences and absences on a grid of hexagons with an equal area (~1.18 km^2^) and shape covering Santa Cruz Island.

To simulate migratory connectivity between the seasonal distributions of tortoises, we used a model called the Optimal Redistribution Simulator (ORSIM), which was shown to capture well‐avian migratory connectivity patterns (Somveille et al., 2021). This model is based on energy optimization and captures two processes: minimizing energetic costs associated with relocating between seasonal grounds, and intraspecific competition for access to energy supply. ORSIM generates origin and destination points for each individual migratory tortoise simulated, and it does so using a solution to the Monge‐Kantorovich transportation problem (Hitchcock, 1941; Rachev, 1984) from linear optimization, which can be formalized with linear programming as follows.

Let $H=\left\{\left(h_{1},k_{h_{1}}\right),\ldots ,\left(h_{m},k_{h_{m}}\right)\right\}$ be the distribution of energy available during the season spent in the highland, where $h_{i}$ is highland site i and $k_{h_{i}}$ is the weight of this site, which corresponds to the energy available at this site; and $L=\left\{\left(l_{1},k_{l_{1}}\right),\ldots ,\left(l_{n},k_{l_{n}}\right)\right\}$ be the distribution of energy available during the season spent in the lowland, with n lowland sites, where $l_{j}$ is lowland site j and $k_{l_{j}}$ is the weight of this site, which corresponds to its energy supply. We want to find a total flow $F=\left[f_{\mathrm{ij}}\right]$, with $f_{\mathrm{ij}}$ the flow of energy between $h_{i}$ and $l_{j}$, that minimizes the overall cost

$$C\left(H,L,F\right)=\sum_{i=1}^{m}\sum_{j=1}^{n}c_{\mathrm{ij}}f_{\mathrm{ij}}$$

where $c_{\mathrm{ij}}$ is the energetic cost associated with relocating between sites $h_{i}$ and $l_{j}$. This function is subject to the following constraints:

(1) $$f_{\mathrm{ij}}\geq 01\leq i\leq m,1\leq j\leq n$$

(2) $$\sum_{j=1}^{n}f_{\mathrm{ij}}\leq k_{h_{i}}\;1\leq i\leq m$$

(3) $$\sum_{i=1}^{m}f_{\mathrm{ij}}\leq k_{l_{j}}\;1\leq j\leq n$$

(4) $$\sum_{i=1}^{m}\sum_{j=1}^{n}f_{\mathrm{ij}}=\min\left(\sum_{i=1}^{m}k_{h_{i}},\sum_{j=1}^{n}k_{l_{j}}\right)$$

Constraint (1) allows energy to move from H to L and not vice versa. Constraint (2) limits the amount of energy that can move away from the highland sites in H and reflects the energy demand of departing individuals. Constraint (3) limits the lowland sites in L to receive no more energy than their energy supply. Finally, constraint (4) specifies that the total amount of energy must be equal to either the total energy demand of highland sites or the total energy supply of lowland sites, whichever one is the smallest, thus forcing it to move the maximum amount of energy possible.

This modeling framework assumes that energy is transferred between highland and lowland sites via migrating tortoises, which are energetically equivalent, i.e., they all have the same energetic needs and cost function, and the same competitive ability. Energy availability across the highland and lowland distributions of tortoises was estimated using NDVI values (see Section 2.1.1). We rescaled these NDVI values so that a total of 1000 migrating individuals were generated by the model. For a complete description of ORSIM including more details about the underlying assumptions see Somveille et al. (2021).

The transportation problem from linear optimization described above can be solved using the transportation‐simplex method (Hillier & Lieberman, 1990), which is implemented in the Earth Mover's Distance (EMD) algorithm (Rubner et al., 2000). The EMD computes the distance between two distributions that minimize the cost of transforming one into the other. To simulate migratory connectivity, we used FastEMD (Pele & Werman, 2008, 2009), which is implemented in the Python wrapper PyEMD. No distance threshold was used when running FastEMD.

#### 2.2.3 Simulating migration trajectories

We simulated the explicit migration trajectories of migrating tortoises between their starting point in the highland and the respective destinations in the lowland that are generated by the migratory connectivity model (Section 2.2.2). To do so, we used the empirical Random Trajectory Generator (eRTG; Technitis et al., 2015) in the R environment. This algorithm generates the movement between two endpoints with a fixed number of steps, which retains the geometric characteristics of real observed trajectories based on tracking data (see detailed description in Kleyheeg et al., 2019; van Toor et al., 2019). The eRTG is similar to a biased correlated random walk and can be best described as a mean‐reverting Ornstein‐Uhlenbeck process (Smouse et al., 2010). The algorithm uses empirical tracking data as a template and takes empirical distributions of the following characteristics of animal movement: step length, turning angles, their autocorrelation at a lag of one step, and the covariance of step length and turning angle. We estimated the distributions of these movement characteristics using the empirical tracking data on tortoises' downslope migratory movement described above, which were thinned to one point per day as we assumed that tortoises stop at one location per day during migration where they eat and produce excrements.

Using the eRTG, we simulated the movement trajectories of the 1000 migrating tortoises, whose origin and destination points were generated by the model of migratory connectivity. For each simulated migrating tortoise, we used the following procedure: We randomly selected an empirical track to configure the eRTG and ran the simulation; if the model converged, we selected the resulting simulated trajectory, but if the model did not converge, we randomly selected another empirical track an ran the eRTG again, continuing until the model converged. All models (i.e., for each simulated individual) ultimately converged. These simulations are stochastic, we therefore repeated this overall procedure 25 times, i.e., each time simulating 1000 migrating tortoises.

#### 2.2.4 Simulating seed dispersal

To simulate the spread of guava seeds by migrating tortoises, we combined the simulated migration trajectories with an empirically informed simulator of tortoises' consumption and excretion of guava seeds. In our model, tortoises are assumed to engage in migratory movement once a day and conversely stop at one location per day during migration. We ran a sensitivity analysis in which tortoises stop at two and four locations per day by interpolating the simulated trajectories. When they are not migrating, tortoises are assumed to stay put, eat and produce excrements. When not migrating, if a tortoise is located where guava is present, we assumed that it eats guava. In addition, each tortoise has a probability of excreting guava seeds based on gut retention time and the results of previous feeding events, determined as:

$$P_{g}=\frac{\sum_{d=1}^{d_{\max}}R\left(d\right)∙F_{d}}{\sum_{d=1}^{d_{\max}}R\left(d\right)}$$

with $F_{d}$: binary result of foraging event at day d before focal time (i.e., 1 if guava was eaten that day and 0 if not); R: probability of excreting seeds from guava eaten at day d based on gut retention time distribution, which was estimated based on results of feeding trials (Sadeghayobi et al., 2011) as a right‐skewed normal distribution with μ=12, σ=8 and xi=2—for example, if guava was eaten at day 5 before focal time, then the probability of excreting guava is $R\left(5\right)=0.05378286$. $P_{g}$ is then the overall probability of excreting guava seeds obtained based on summing the probabilities for each day up to $d_{\max}$, which indicates a threshold, which was set to 28 days (Sadeghayobi et al., 2011), above which guava seeds were considered to be no longer present in the gut. For each of the 28 days preceding the start of the simulation, a tortoise was set to have eaten guava given probability = 0.21, which corresponds to the proportion of the highland distribution that is covered by the spatial distribution of guava.

To determine whether guava is excreted when a tortoise is not migrating, we sampled a value between 0 (no guava excreted) and 1 (guava excreted) based on $P_{g}$. Then, if guava is excreted, the number of guava seeds excreted during the excretion event is determined by randomly sampling an empirically‐estimated truncated normal distribution with μ=1443 and σ=2057 (Ellis‐Soto et al., 2017). Using this methodology, we modeled where and how many guava seeds are excreted along the migratory trajectory of each simulated migrating tortoises, which we call “seed rain.” We then overlaid the seed rain on the projection of the SDM that predicts establishment success (i.e., binary value of successful versus nonsuccessful establishment, based on Ellis‐Soto et al., 2017; see Section 2.1.1) in order to determine whether seeds are likely to germinate or not (i.e., seed fate).

### 2.3 Model validation

To validate the seed dispersal prediction of our model, we used a dataset of collected dung piles containing guava seeds from Ellis‐Soto et al. (2017) and added a few others opportunistically collected since then. The dataset contains 222 dung piles, including 101 that are containing guava across an elevation gradient from 28 to 419 m. To better understand the spatial context in which tortoise‐dispersed guava seeds are deposited across our study area, we associated dung pile occurrences with landcover classes of Santa Cruz Island from Rivas‐Torres et al., 2018. We focused specifically on an area locally known as “La Reserva” to the south and southwest of the island, which is the core distribution area of *C. porteri* (Ellis‐Soto et al., 2017). We extracted landcover information at the location of the observed and simulated dung piles containing guava. We inspected whether the frequency density of dung piles containing guava seeds across elevation and the distribution of dung piles containing guava seeds across habitats, in particular in agricultural areas versus the Galapagos National Park, were similar for simulated versus observed dung piles.

### 2.4 Seed fate

To estimate whether guava seeds can establish and become adult reproductive trees, we made use of a species distribution model (SDM) for guava previously published (Ellis‐Soto et al., 2017). This model identifies suitable habitat for the establishment of guava (seed dispersal efficiency) by relating environmental predictors, i.e., temperature and precipitation long‐term averages and seasonality commonly referred to as bioclimatic variables (Hijmans et al., 2005), to empirical georeferenced guava locations. A threshold was chosen to convert continuous suitability values outputted by the model to binary establishment and persistence success based on the minimum climatic suitability in which an actual guava plant was observed during a vegetation survey across Santa Cruz Island. This SDM thus provides a proxy of seed fate for seeds deposited across a gradient of climatic suitability.

## 3 RESULTS

### 3.1 Simulated and observed seed dispersal by migratory tortoises

**FIGURE 2:**
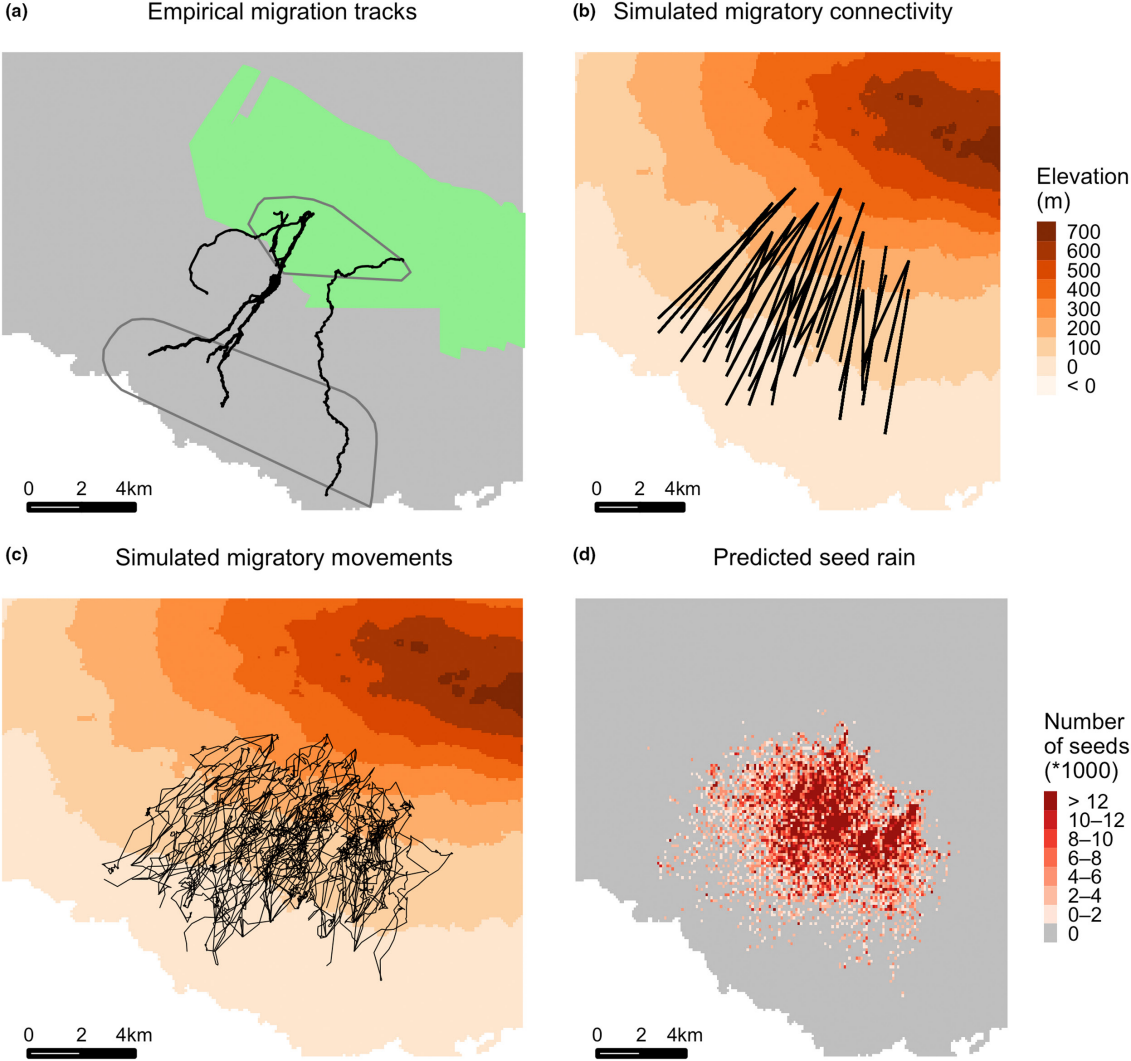
Tortoise migrations and guava seed rain. (a) Empirical migration tracks used to calibrate the trajectory simulator eRTG; in green is the agricultural zone and in gray is the Galapagos National Park; polygons with gray borders indicate the highland and lowland distribution estimated for the focal tortoise population. (b) Simulated migratory connectivity outputted from ORSIM, which connects highland and lowland sites. (c) Simulated downslope migration trajectories of tortoises, from highland to lowland; black lines indicate the migration trajectories of 100 randomly sampled simulated individuals; (b) density of guava seeds dispersed by simulated tortoises migrating downslope, also called “seed rain”.

**FIGURE 3:**
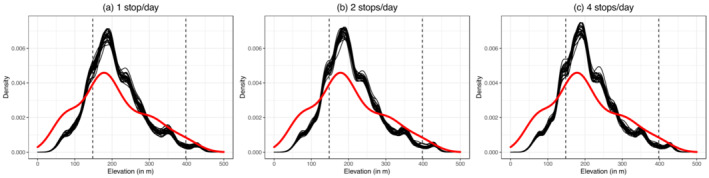
Model simulation captures empirical dispersal of guava seeds by migrating tortoises. Distribution of observed (red) and simulated (black) tortoise dung piles with guava across the elevational gradient of Santa Cruz Island for simulations with stops at (a) 1 location per day, (b) 2 locations per day, and (c) 4 locations per day, where simulated tortoises eat and produce excrements. Black lines indicate the results of the 25 different simulations and thus exhibit the stochastic variation in simulation output.

Our data‐driven modeling framework was able to simulate the downslope migration of adult male Galapagos tortoises between highland and lowland seasonal grounds on Santa Cruz Island (Figure 2). The vast majority of trajectories and the overall migration pattern of the population simulated by the model appear realistic when visually compared with empirical tracks. The resulting dispersal of guava seeds by migrating tortoises spreads from the highland distribution to the lowland distribution, decreasing in intensity as tortoises arrive closer to the lowlands (Figure 2d). This pattern of the density of dung piles with guava seeds across the elevational gradient of Santa Cruz is congruent with empirical dung piles data (Figure 3a; two‐sample Kolmogorov–Smirnov test combining all simulation runs together: *D* = 0.13641, *p* = .05223), thus validating the model prediction. The occurrences of observed and simulated dung piles containing guava both peak at ca. 180 m in elevation, with a decreasing density towards lower (Galapagos National Park areas) and higher (Agricultural and privately owned areas) elevations (Figure 3a). However, simulated guava seeds appear to be somewhat less frequent than observed at lower elevations, ca. 100 m (Figure 3a). Both observed and simulated dung piles containing guava seeds are outside the current elevational range of guava in Santa Cruz Island, potentially facilitating the expansion of this invasive species (Figure 3). The sensitivity analysis indicates that the number of times migratory tortoises stop to feed and excrete per day does not appear to affect the results (Figure 3; two‐sample Kolmogorov–Smirnov test combining all simulation runs together comparing simulation results for 1 stop per day with (i) simulation results for 2 stops per day: D = 0.00705, *p* < .001; and 4 stops per day: D = 0.00962, *p* < .001).

### 3.2 Estimating seed fate using landcover and SDMs

By combining the guava seed rain simulated from our model (Figure 2d) with landcover categories, we provide a spatial and ecological context in which guava seeds were deposited. Simulated guava seeds were deposited in five different landcover classes, with a pattern of deposition in these different landcover classes that largely matches the one from empirical data (Figure S1). Guava seeds were deposited mostly in agricultural areas but also reached the Galapagos National Park (Figure S2). Some discrepancies exist between simulation and empirical data, notably that the model predicts guava seeds to be deposited substantially more than suggested from empirical data in areas occupied by invasive species but less than empirical data suggest in evergreen forests and shrubs (Figure S1). In addition, when applying our threshold guava SDM mask, we were able to create a spatially‐explicit prediction of successful seed dispersal for *C. porteri*, simulating guava seeds with the potential to germinate and establish under current climatic conditions (Figure S3).

## 4 DISCUSSION

In this study, we developed a modeling framework for simulating the dispersal and fate of propagules carried by migratory animals. In contrast to the previous work that used spatially‐homogeneous dispersal kernels (Bullock et al., 2017; Nathan, 2006; Nathan et al., 2012; Pires et al., 2018) or daily foraging movement models (Beaune et al., 2015; Campos‐Arveiz et al., 2008; Kleyheeg et al., 2017; Olesky et al., 2017; van Toor et al., 2019; Wright et al., 2020), we model animal‐mediated seed dispersal by simulating individual‐level directional migratory movement explicitly. It provides a quantitative framework for predicting long‐distance propagule dispersal events by animals, which have rarely been quantitatively predicted despite their ecological importance (Nathan & Muller‐Landau, 2000). Kleyheeg et al. (2019) modeled how a seasonally migrating local population of mallards spreads seeds over long distances, but this was done for a single departure location while our model, via the ORSIM component (Figure 1), simulates seed dispersal by migratory animals from multiple populations simultaneously across space, thus generalizing the approach for studying entire species and opening up potential applications. Our framework also builds on previous work that developed modular models able to be calibrated by empirical data while going a step further by being fully data‐driven at every step of the modeling process from population seasonal distributions to gut retention time to seed fate. Our model is able to predict for the first time the magnitude and direction under which a migratory animal population distributed across space disperses plant species into novel habitats, and quantify how the seasonal migration of herbivores may be a major seed dispersal vector and expand the distribution range of invasive plant species.

We applied our modeling framework to guava seed dispersal by Galapagos tortoises (*C. porteri*) on Santa Cruz Island, which is a data‐rich system with relevance for conservation. Our model has the good predictive ability for the pattern of spread of guava seeds by migrating tortoises (Figure 3) and can therefore be used for making predictions beyond the individuals and areas where data were collected. We predicted clusters of heavy seed dispersal alongside tortoises' downslope migration range (Figure 2d) and identified areas most likely to see establishment success (Figure S3). We found that Galapagos tortoises' seed dispersal may particularly contribute to guava downslope range expansion on Santa Cruz Island, due to both long gut retention time and tortoise's long‐distance migration across vegetation zones. In particular, we predict that tortoises are dispersing a significant amount of guava seeds into the Galapagos National Park, which has important consequences for the native flora as guava is an invasive species that has already altered natural ecosystems on the Galapagos (Weber, 2003; Wiggins & Porter, 1971) and is threatening local and endemic plant species (de Lourdes Torres & Mena, 2018). Our results also suggest that the frequency at which behavior (i.e., migratory movements, eating, excreting) is repeated during the migration season does not have an important role to play in predicting long‐distance dispersal events (Figure 3).

Our framework is flexible and modular as it allows for increasing complexity and the integration of multiple data sources (Figure 1). For example, if the number of seeds excreted and establishment success is unknown for a study system, this information could be omitted and the model could simply predict the location of seed dispersal events across space. More information on population distribution and individual‐level movement, which is increasing exponentially thanks to advances in citizen science and tracking technology (Kays et al., 2020), could be used to calibrate more accurately the animal migration module (i.e., model components UD, ORSIM, and eRTG; Figure 1). In addition, besides using SDMs, other proxies for seed fate such as additional landcover information or microclimate based on fine‐scale topography could be employed (Leempoel et al., 2015; Maclean et al., 2019; van Toor et al., 2019). In situ germination trials of seeds along environmental gradients would provide the greatest insights into the seed dispersal efficiency of a migratory animal species, which is relevant as plant mortality depends on environmental conditions and is highest when plants are seedlings (Terborgh et al., 2014). Future studies could also include plant demography in addition to the movement ecology of seeds in order to fully model the contribution of animal‐mediated seed dispersal to the range expansion of an invasive species (Beckman et al., 2019). Multiple plant species could also be considered simultaneously in this modeling framework, which could therefore be used to predict the contribution of a migratory animal species to changes in plant community composition across an elevational or latitudinal gradient.

A research avenue for which our modeling framework could provide an important contribution is investigating the impact of environmental change on ecosystem processes. In particular, change in climate and habitat quality might alter animal migration patterns and seed germination success, thus affecting animal‐mediated seed dispersal. The model integrates models of animal movement influenced by the distribution of resources (using NDVI here but other data could be used) and plant establishment success based on climate suitability from species distribution modeling (Ellis‐Soto et al., 2017), with the availability of future climate projections and predicted change in resource supply, our approach could be used to make future predictions for where will seeds disperse and successfully establish under various scenarios of environmental change. Thus, the predictive ability and flexibility of our framework make it a valuable tool for investigating seed dispersal under climate change, which cannot be easily predicted using existing modeling approaches.

Our framework can be applied to a wide range of systems where ecosystem processes are affected by migratory animal species. It would be interesting for example to investigate how migratory populations living in different environmental settings would spread an invasive plant in different ways, such as, for example, how different populations of tortoises spread guava on different islands in the Galapagos. Such predictions would allow identifying which islands and ecosystems are most at risk of invasion by guava and it would therefore inform where to focus conservation efforts. In addition, our modeling framework could be used to investigate the relative roles of different migratory animal species, given their movement ability and patterns, in spreading the seeds of an invasive plant throughout an ecosystem, which is a promising research avenue with important consequences for conservation and ecosystem functioning. In the case study presented here, giant tortoises are not the only animals capable of dispersing seeds across Galapagos Islands. Galapagos mockingbirds (*Mimus parvulus*), Darwin finches (Geospiza spp.), and introduced cattle, pigs, and goats also consume guava, but they move shorter distances (Buddenhagen & Jewell, 2006) and cross‐less from agricultural areas in the highlands to the national park in the lowlands when compared to tortoises. Humans in the Galapagos are also potential long‐distance dispersers of guava (Auffret et al., 2014). It would be informative to adapt our model, in particular the animal movement component, to quantify the relative contributions of these different vectors, which are mobile but not necessary seasonal migrants, to the spread of guava. Finally, our modeling framework could also be used to investigate ecosystem processes other than seed dispersal. For example, it would be possible to apply it to quantitatively model how migratory animals affect nutrient transport across ecosystems, which can be quantified either through empirical measurements of excretion rate or metabolic measurements. These different applications can improve our understanding on how animals connect ecosystems and landscapes across spatiotemporal scales and have important conservation implications for the management of ecosystems that include migratory animals.

## AUTHOR CONTRIBUTIONS

**Diego Soto:** Conceptualization (equal); data curation (equal); formal analysis (supporting); investigation (equal); methodology (equal); writing – original draft (equal); writing – review and editing (equal).

## CONFLICT OF INTEREST

All authors claim no conflict of interest.

### OPEN RESEARCH BADGES

This article has earned Open Data and Open Materials badges. Data and materials are available at https://doi.org/10.5441/001/1.6gr485fk.

## Supporting information


Figure S1.
Figure S2.
Figure S3.

## Data Availability

Land cover classification maps are available on the supplementary material of Rivas‐Torres et al. (2018), and guava distribution in Santa Cruz Island is available on the supplementary material of Laso et al. (2019). Animal tracking data used in this manuscript are available under creative comma zero licensing. Galápagos giant tortoise (*C. porteri*)–tracking data are available at https://www.datarepository.movebank.org/handle/10255/move.834 (10.5441/001/1.6gr485fk; Bastille‐Rousseau et al., 2019). The computer code used for this study is available at https://github.com/msomveille/galapagos‐tortoises.git.
