# Supplementary material for: Linking animal migration and ecosystem processes: Data‐driven simulation of propagule dispersal by migratory herbivores
Source: Ecol Evol. 2022 Oct 18;12(10):e9383. doi: 10.1002/ece3.9383 (PMC9577414; doi:10.1002/ece3.9383)
Supplement: Supplementary file 1 — Figure S1. Figure S2. Figure S3. [file ECE3-12-e9383-s001.docx]

1. **Supplementary Material**

**
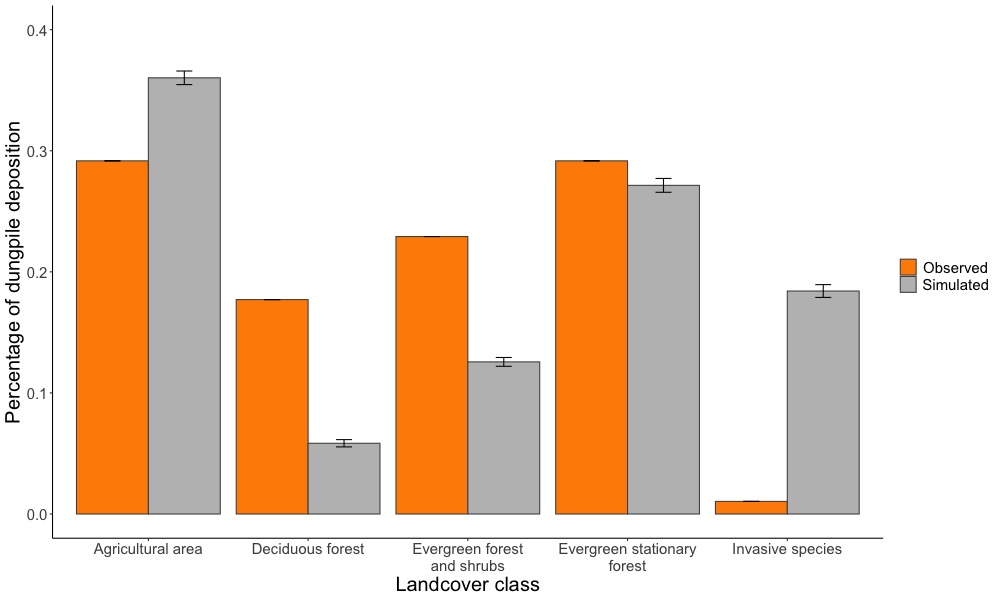
**

**Figure S1. Landcover types under which simulated guava seeds are deposited.** Spatial deposition of observed (orange) and simulated (grey) dung piles containing guava for different landcover classes from Rivas-Torres *et al.* (2018a). The grey bars indicate the mean value among the 25 simulation runs, and the error bars indicate the stochastic variation in simulation output.

**Figure S2. Model simulation captures empirical dispersal of guava seeds by migrating tortoises into the Galapagos National Park.** Observed (a) and simulated (b) guava seed deposition into the agricultural and national park in Santa Cruz, Galapagos.


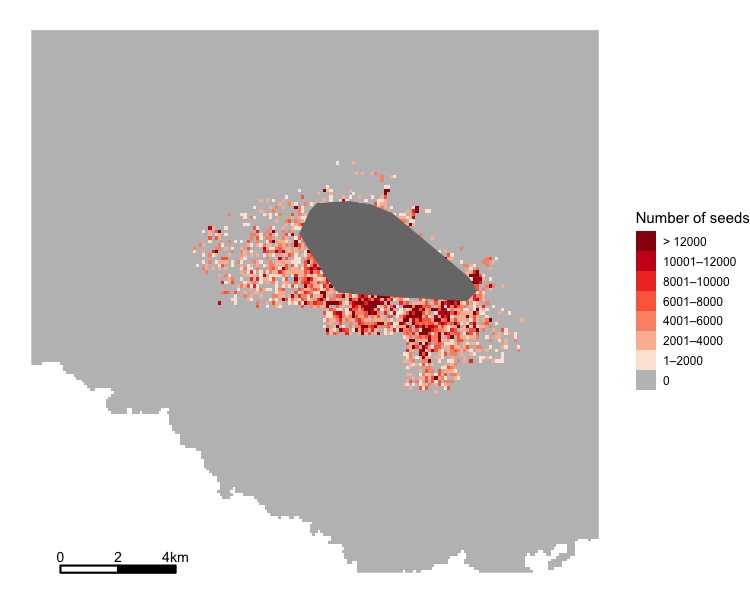


**Figure S3. Estimated successful seed dispersal.** Density of germinated guava seeds dispersed by simulated migrating tortoises obtained by combining the simulated seed rain outputted by one simulation run with the establishment success of guava based on a species distribution model (Ellis-Soto *et al.* 2017). In dark grey: highland distribution of the population.
